# Dose Rationale for Amoxicillin in Neonatal Sepsis When Referral Is Not Possible

**DOI:** 10.3389/fphar.2020.521933

**Published:** 2020-09-25

**Authors:** Salvatore D’Agate, Flora Tshinanu Musuamba, Oscar Della Pasqua

**Affiliations:** Clinical Pharmacology and Therapeutics Group, University College London, London, United Kingdom

**Keywords:** amoxicillin, sepsis, pediatrics (drugs and medicines), dose optimization, newborn, infants, pharmacokinetic-pharmacodynamic, population pharmacokinetic analysis

## Abstract

**Background:**

Despite the widespread use of amoxicillin in young children, efforts to establish the feasibility of simplified dosing regimens in resource-limited settings have relied upon empirical evidence of efficacy. Given the antibacterial profile of beta-lactams, understanding of the determinants of pharmacokinetic variability may provide a more robust guidance for the selection of a suitable regimen. Here we propose a simplified dosing regimen based on pharmacokinetic-pharmacodynamic principles, taking into account the impact of growth, renal maturation and disease processes on the systemic exposure to amoxicillin.

**Materials and Methods:**

A meta-analytical modeling approach was applied to allow the adaptation of an existing pharmacokinetic model for amoxicillin in critically ill adults. Model parameterization was based on allometric concepts, including a maturation function. Clinical trial simulations were then performed to characterize exposure, as defined by secondary pharmacokinetic parameters (AUC, C_max_, C_min_) and T>MIC. The maximization of the T>MIC was used as criterion for the purpose of this analysis and results compared to current WHO guidelines.

**Results:**

A two-compartment model with first order absorption and elimination was found to best describe the pharmacokinetics of amoxicillin in the target population. In addition to the changes in clearance and volume distribution associated with demographic covariates, our results show that sepsis alters drug distribution, leading to lower amoxicillin levels and longer half-life as compared to non-systemic disease conditions. In contrast to the current WHO guidelines, our analysis reveals that amoxicillin can be used as a fixed dose regimen including two weight bands: 125 mg b.i.d. for patients with body weight < 4.0 kg and 250 mg b.i.d. for patients with body weight ≥ 4.0 kg.

**Conclusions:**

In addition to the effect of developmental growth and renal maturation, sepsis also alters drug disposition. The use of a model-based approach enabled the integration of these factors when defining the dose rationale for amoxicillin. A simplified weight-banded dosing regimen should be considered for neonates and young infants with sepsis when referral is not possible.

## Introduction

In 2015, the World Health Organization (WHO) published new guidelines for the management of possible serious bacterial infection in young infants when referral is not feasible (World Health Organization, 2015a). These guidelines are based on increasing evidence, which shows that in resource-limited settings many young infants with signs of severe infection are treated sub-optimally, as compared to those receiving treatment in a hospital setting. In fact, multiple factors have been identified, which contribute to sub-optimal or inadequate treatment of these vulnerable patients, including accessibility, acceptability and affordability (Baqui et al., 2008; Zaidi et al., 2012; African Neonatal Sepsis Trial et al., 2015). Consequently, in the absence of referral, these cases result in unnecessary, potentially preventable infection-related new-born deaths.

While access to referral facilities should be promoted for the treatment of severe infections, the effectiveness of community-based interventions clearly depends on the availability of suitable, affordable pharmaceutical dosage forms and simplified dosing regimens. Indeed, numerous clinical trials have been performed over the last few years to assess the efficacy of different doses of amoxicillin and penicillin-gentamicin combination in resource-constrained settings (Baqui et al., 2013; Group, 2013; Zaidi et al., 2013a; Mir et al., 2017). The individual and combined results of these trials show that the selected antibiotic regimens for neonatal sepsis (which include oral amoxicillin) are equally effective when compared with the standard 7-day course of injectable penicillin and gentamicin (Nair, 2017).

Amoxicillin has been included into the WHO’s List of Essential Medicines and is considered one of the most effective and safe medicines needed in a health system (World Health Organization, 2015b). However, irrespective of the comparable efficacy observed for different regimens in controlled clinical trials, the dose of amoxicillin administered to patients younger than 3 months of age has been selected in a rather empirical manner. Similarly, the current WHO guidelines (World Health Organization, 2015a), which recommend a dose of 50 mg/kg twice daily for managing possible serious bacterial infection in young infants 0–59 days old when families do not accept or cannot access referral care seem to rely on the same principles, i.e., empirical evidence of efficacy. Such an approach does not warrant optimal exposure of new-borns to antibiotic agents, in whom pharmacokinetics and drug disposition is known to differ significantly from older children and adults. Moreover, the use of doses linearly corrected by body weight (i.e., mg/kg) often overlooks the effect of covariate factors on the pharmacokinetics of most drugs in this age group.

Understanding of the pharmacokinetics and pharmacodynamics of amoxicillin is therefore essential to optimize and simplify the dosing regimen in new-borns with severe bacterial infection. Here, we apply quantitative clinical pharmacology methods, and more specifically modeling and clinical trial simulations to identify a simplified regimen of amoxicillin taking into account pharmacokinetic-pharmacodynamic (PKPD) concepts.

From a clinical pharmacology perspective, it has been established that for β-lactams, the PKPD index that best correlates with efficacy is the duration that the unbound concentration exceeds the MIC, expressed as a percentage of the dosing interval (% T>MIC). Whereas the pharmacodynamic target appears to be different for each β-lactam group (penicillins, cephalosporins, and carbapenems), it is often assumed that exposure levels associated with T>MIC of at least 40% are required for amoxicillin (de Hoog et al., 2005; Downes et al., 2014). This target will obviously vary depending on the etiology and severity of the infection. Another point to consider when defining the dose rationale for a drug is the post-antibiotic effect (PAE). PAE refers to the continued suppression of bacterial growth for prolonged periods when drug concentrations fall below the MIC of the bacteria (Craig and Ebert, 1990). β-Lactams demonstrate a modest PAE against Gram-positive organisms, but no PAE (except carbapenems) against Gram-negative organisms (Craig and Ebert, 1990; Rodvold, 2001). Based on the aforementioned concepts, it can be anticipated that dose levels that do not ensure pharmacodynamic target attainment are likely to lead to clinical failure and selection of resistant strains (Craig and Ebert, 1990; Rodvold, 2001; Drusano, 2003; Drusano, 2007).

Accordingly, any attempt to simplify dosing regimens will need to take into account all relevant factors known to affect the pharmacokinetics of the drug of interest. In the case of neonatal sepsis and severe bacterial infections, differences in organ function and disease-related changes in hemodynamics and vascular permeability can have major impact on drug disposition (West et al., 1948; Arant, 1978; Jones and Chesney, 1992; Blackburn, 1994; Samardzic et al., 2016). However, despite the wide use of oral amoxicillin and evidence of its efficacy in resource-limited settings, knowledge of its pharmacokinetics in the neonatal patient population is limited. In particular, a more quantitative evaluation to assess the impact of age-related changes on the disposition of amoxicillin is lacking in both pre-term and term new-borns.

From a pharmacokinetic perspective, amoxicillin does not undergo biotransformation and metabolism. Given its physicochemical properties (high water solubility and low plasma protein binding), amoxicillin is readily excreted by glomerular filtration (Todd and Benfield, 1990). Additionally, there is evidence for the contribution of renal tubular secretion to the total renal clearance of amoxicillin, since it has been shown that concomitant administration of probenecid reduces its renal clearance (Staniforth et al., 1983). As glomerular filtration rate and tubular secretion in neonates are subject to maturation processes, it can be anticipated that dose selection in neonates needs to account for such covariates. Furthermore, available data in critically ill patients suggest that pharmacokinetic disposition can be significantly altered as sepsis symptoms worsen (Zamoner et al., 2016).

Based on pharmacokinetic bridging and extrapolation principles (Cella et al., 2010a; Cella et al., 2010b), we explore the possibility of deriving simplified fixed dose regimens for oral amoxicillin, which provide comparable exposure irrespective of age or disease severity. To this purpose, a population pharmacokinetic model was developed taking into account the role of maturation in renal function and the effect of disease on systemic drug levels. Using clinical trials simulations, simplified regimens were subsequently identified, which maximize T>MIC.

## Materials and Methods

**Figure 1:**
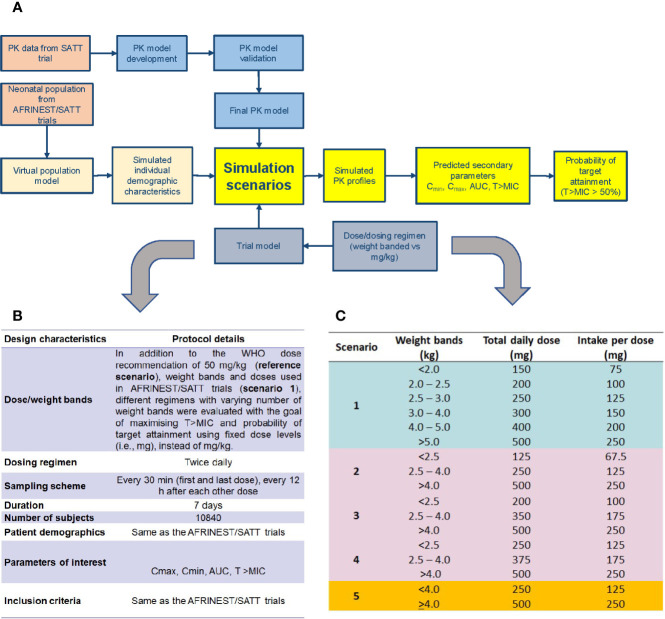
**(A)** Workflow for the implementation of clinical trial simulations aimed at the evaluation of simplified dosing regimens for amoxicillin in neonatal sepsis; **(B)** design characteristics for the identification of a simplified dosing regimen using clinical trial simulations; **(C)** simulation scenarios evaluated for the identification of a simplified dosing regimen based on weight bands and fixed doses.

The different steps and procedures associated with model development and evaluation, as well as the subsequent use of model parameters in clinical trial simulations aimed at dose optimization and feasibility of simplified dosing regimens are summarized in a workflow diagram in **Figure 1A**. Details on each of the steps and procedures are described in the subsequent paragraphs.

### Clinical Data

**Figure 2:**
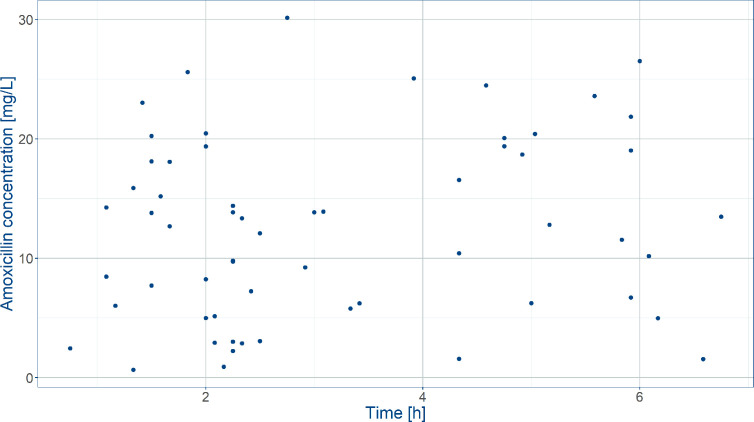
Observed amoxicillin concentrations in the SATT trial (Mir, 2013) after weight‐banded dosing in neonates. Blood was sampled between 0 and 8 h after the first dose on the first treatment day.

Demographic data and efficacy information from patients who were enrolled into the AFRINEST and SATT trials were used as reference for the purpose of the current analysis. Baseline demographic characteristics were complemented by pharmacokinetic data from a subset of the patients enrolled into the SATT trial, who contributed with sparse blood sampling for further characterization of the exposure to amoxicillin in this population (**Table 1**) (Mir, 2013). In this study, blood samples were obtained from each participant prior to amoxicillin administration, 2 to 3 h post-dose, and 6 to 8 h post-dose (**Figure 2**).

**Table 1:** Demographic characteristics of the patient enrolled into the pharmacokinetic group (SATT study) (Mir, 2013).

Patient characteristics	Median (range)
Number of patients	44
Age (days)	11.5 (1 – 56)
Weight (kg)	2.84 (1.7 – 5)
Gestational age (weeks)	38 (34 – 40)
Male, %	55

The individual demographic data from both trials were used to develop a population model and subsequently simulate a virtual cohort of patients with similar characteristics of the neonatal sepsis patients. As shown in **Figure S1**, accurate assessment of the effects of baseline demographic characteristics on drug disposition needs to take into account the skewed distribution of post-natal age and body weight, which reflects the incidence and prevalence characteristics of sepsis after birth. In the same way, individual amoxicillin concentrations were used to evaluate the pharmacokinetics and assess the effect of disease on drug disposition parameters. Subsequently, simulation scenarios including different dosing regimens were considered taking into account the individual demographic characteristics. Given the impact of premature birth on organ function and drug disposition, weight, post-natal age, birth date and gender were complemented by predicted post-natal age using the approach described by Sumpter and Holford (2011). The method relies on the assumption of a correlation between actual body weight, weight at birth and gestational/post-natal age.

Further details of the trial protocols are available elsewhere (Zaidi et al., 2013b; African Neonatal Sepsis Trial et al., 2015; Baqui et al., 2015). All studies included in the current analysis have been conducted in full conformance with the principles of the Declaration of Helsinki and with the local laws and regulations concerning clinical trials.

### Pharmacokinetic Model

As the pharmacokinetics of amoxicillin has not been previously described in neonatal sepsis patients, the population pharmacokinetic model developed by Carlier et al. (2013) for critically ill adults (sex: 85% male, interquartile range age: 58-72 years, weight: 70-79 kg) was selected to describe drug disposition in neonates.

In contrast to traditional modeling procedures, where model selection and parameter identification are primarily data driven, here we have relied on extrapolation principles to derive renal clearance based on amikacin, whereas allometry was used to scale the volume of distribution and inter-compartmental clearance. First, the model was re-parameterized to allow for allometric scaling of the central and peripheral volume of distribution and inter-compartmental clearance in neonates and young infants. A fixed exponent of 1 was used for volume of distribution and 0.75 for inter-compartmental clearance. Renal clearance in neonates was then assumed to be affected not only by differences in organ size, but also by maturation processes. An attempt was made to distinguish changes in renal function due to the underlying maturation processes from drug specific characteristics, such as elimination by active transport mechanisms. The distinction between system- and drug-specific properties has been explored by different research groups. Here we applied the approach proposed by Zhao and colleagues (Zhao et al., 2013), who have identified a general maturation function to predict the clearance of different drugs with known renal elimination in neonates (gestational age: 17‐42 week; postnatal age: 1 – 120 days).

Even though organ function in sepsis patients can deteriorate relatively fast, we have assumed that maturation would be comparable across age-matched groups. Amoxicillin clearance (CL_amox_) was extrapolated using amikacin parameters as a reference compound, based on the following relationship:

1) $$CL_{amox}=CL_{amik}\cdot \frac{fu_{amox}}{fu_{amik}}$$

where

2) $$CL_{amik}=CL_{amik,pop}\cdot {\left(\frac{BW}{1.75}\right)}^{1.34}\cdot \left(1+0.213\cdot \frac{PNA}{2}\right)$$

and

3) $$fu_{drug}=\frac{1}{1+\frac{(1-fu_{adult})\cdot P_{neonate}}{fu_{adult}\cdot P_{neonate}}}$$

CL_amik_ and CL_amik,pop_ are the individual and population clearance estimates of amikacin, respectively, BW is the weight at birth, PNA is the post-natal age, fu is the unbound fraction, P is the plasma protein concentration.

The aforementioned steps provided the initial population parameter estimates describing the disposition of amoxicillin. This information was subsequently used along with sparse pharmacokinetic data from the SATT trial (Mir, 2013) (**Table 1**) with the objective of refining model parameter estimates and ensuring accurate characterization of the effect of sepsis, as well as the potential impact of different formulations on drug absorption. In brief, the pharmacokinetics of amoxicillin was best described by a two-compartment model with first order absorption and first order elimination (**Figure S2**). Bioavailability was fixed to previously published literature values (Weingartner et al., 1977).

On the other hand, thanks to the availability of sparse pharmacokinetic data, estimates of the absorption rate constant could also be derived for this patient population. In addition, it was possible to incorporate the effect of sepsis on the volume of distribution, according to the formulas below:

4) $$V_{c}=V_{c,pop}\cdot \left(\frac{WT}{75}+Dis\right)$$

5) $$V_{p}=V_{p,pop}\cdot \left(\frac{WT}{75}+Dis\right)$$

where V_c_, V_c,pop_, V_p_, V_p,pop_ are the individual and population estimates of the central and peripheral volumes of distribution, respectively, WT is the current body weight and *Dis* is the effect of the disease on drug distribution.

Inter-individual variability (IIV) on clearance and volume of distribution was estimated from the available paediatric data in combination with prior parameter distributions based on the estimates obtained by Carlier and colleagues (Carlier et al., 2013). Inter-occasion variability (IOV) was also identified for volume of distribution. The inclusion of IOV allowed us to account for the variability associated with the differences in drug distribution during the course of treatment. The effect of disease was assumed to be time-variant and change nonlinearly during the course of treatment until remission of the symptoms:

6) $$Dis={\text{Dis}}_{max}\cdot \left(1-\frac{t^{5}}{72^{5}+t^{5}}\right)$$

where *Dis*_max_ represents the maximum effect of sepsis on the volume of distribution, t is the time since onset of treatment, 72 h is the time associated with half of the maximum effect, 5 is the slope parameter value. All subjects were considered to have responded to treatment to allow the estimation of a parameter relative to the effect of the disease on volume of distribution.

Model building criteria included successful minimization, standard error of estimates and termination of the covariance step. Comparison of hierarchical models was based on the changes to the objective function value (OFV). Changes in the OFV after inclusion of a parameter approximate a χ^2^ distribution with one degree of freedom. A parameter was considered statistically significant (p<0.05) and added into the model if changes to OFV were greater than 3.84. As allometric scaling was applied to extrapolate parameters across populations, the implementation of a stepwise covariate inclusion and exclusion procedure was not deemed necessary. Outlier detection was initially based on visual examination of individual and study variables. Furthermore, data points identified with conditional weighted residuals >3 or extreme values (> 3) in a normalized prediction discrepancy error (NPDE) plot were excluded from the analysis during the initial model building process. Goodness-of-fit was assessed by statistical and graphical methods, including population and individual predicted vs. observed concentrations, conditional weighted residuals vs. population predicted concentrations and time. A visual predictive check was utilized to evaluate the adequacy of the final model, including the effects of statistically significant covariates (Yano et al., 2001). Given that the effect of sepsis on drug disposition varies over time and pharmacokinetic data were collected only at the beginning of treatment in the SATT trials, model performance was evaluated using external data, in which the disposition of amoxicillin was investigated at the end of treatment (Weingartner et al., 1977).

### Simulation Assumptions

Six important assumptions are required for the assessment and interpretation of the results from the different simulation scenarios, namely:

1. Variability in treatment response (clinical cure) was assumed to be directly linked to variability in pharmacokinetics, rather than bacterial resistance.
2. To take into account the variability in body weight, each patient was considered to have a weight within the same percentile of the growth curve from birth until completion of the study.
3. Correlations between demographic characteristics and physiological processes associated with drug disposition were treated as constant across the course of disease unless available evidence showed otherwise.
4. The effect of diarrhea and other gastro-intestinal tract symptoms on the overall rate and extent of absorption of amoxicillin was considered to be minimal after oral administration. As such, bioavailability estimates were assumed to be similar across the population. Variability in systemic exposure was therefore assumed to be caused primarily by interindividual differences in disposition parameters, rather than bioavailability. In addition, pharmacokinetics was considered to be linear beyond the observed range of concentrations if higher doses (i.e., up to two-fold) were used in simulation scenarios.
5. For comparison purposes, differences in the simulated scenarios of up to 10% in secondary measures of exposure were not considered as clinically relevant. This threshold was meant to account for model uncertainty. This figure also takes into account current regulatory guidelines for changes in dosage forms.
6. The effect of variable adherence to treatment not included in the current analysis. Dose missingness is therefore assumed to be at random and compliance to be dose-independent in this population.

### Simulation Scenarios: *In Silico* Trial Protocol

Amoxicillin exposure following twice daily oral administration was simulated in a hypothetical population of 10840 children with ages varying from 0 to 59 days, with similar baseline demographic characteristics of the neonatal patients enrolled into the AFRINEST and SATT trials. Further details regarding the study design characteristics used across the different simulation scenarios are summarized in **Figure 1B**. The currently recommended dose by WHO of 50 mg/kg was used as reference for the purpose of comparison between regimens.

The endpoints of interest in our analysis included the plasma concentration vs. time profile, maximum concentration (C_max_), trough concentration (C_min_), area under the curve (AUC), T>MIC and probability of target attainment (PTA) associated with twice daily dosing regimens. The selection of a simplified regimen according to weight bands was based on evidence of comparable exposure across the target population, while maximizing the number of patients with acceptable time above MIC. In addition, an attempt was made to compare the proportion of patients at risk of not reaching at least 50% of the T>MIC after administration of the simplified and recommended WHO dosing regimens.

Given that amoxicillin is delivered orally, and absorption is rapid, formulation was not considered a significant source of variability in the simulation scenarios. Frequency and times for pharmacokinetic sampling were based on a serial sampling scheme for the purposes of estimating AUC over the dosing interval. C_max_ and C_min_ were determined, respectively, by the maximum predicted concentration in each dosing interval and the value of predicted concentration immediately before the next dose. The trapezoidal rule was applied for the integration of the concentration vs. time data and calculation of the AUC, while T>MIC was derived directly from the individual concentration vs. time profiles. Three MIC values, namely 2, 4 and 8 mg/L, were used to account for the natural distribution and variation in bacterial susceptibility (The European Committee on Antimicrobial Susceptibility Testing, 2015).

**Figure 1C** shows the dose and total daily dose evaluated in each scenario. It should be noted that the weight bands included in the current analysis have been restricted to ranges which are aligned with those used for concurrent medications, such as gentamicin. Moreover, where appropriate the proportion of individuals who do not reach at least 50% above the time above MIC was used to rank the suitability of simplified regimens. This threshold is based on historical evidence from microbiological, safety and efficacy studies (Howie et al., 1985; Craig, 1996; Craig, 1998; Dagan, 2003; Pichichero et al., 2008a; Pichichero et al., 2008b). Likewise, peak concentrations were summarized without a strict cut-off or reference value. Consequently, we have decided not to perform any formal statistical hypothesis testing to compare scenarios, but simply to identify the best performing regimens. To that purpose, the probability of target attainment was deemed the most suitable criterion for ranking the performance of the different regimens. Final recommendations were aimed at maximizing the predicted percentage (%) of sepsis patients aged 0 – 59 days with T>MIC above a reference threshold level of 50%.

All simulations were performed using NONMEM version 7.3 (ICON Development Solutions, Ellicott City, MD, USA). R version 3.1.2 (R Core Team, 2013) was used to graphically summarize the results.

## Results

Our analysis shows how empirical dosing recommendations can be assessed in a systematic manner, taking into consideration the contribution of factors known to affect drug disposition in the neonatal patient population. Given the objective of this investigation, first a brief overview is provided of the pharmacokinetic modeling results and parameter estimates which were used across the different scenarios. We then present the simulation results. For the sake of clarity, only two scenarios will be discussed in addition to the selected simplified regimen. The first set refers to the patient population pool from the AFRINEST/SATT studies used for re-sampling of the demographic characteristics across all scenarios. The second refers to the 2015 WHO recommendations for management of possible serious bacterial infections in young infants 0-59 days old when referral care is not possible. An overview of the demographic variables included in the clinical trial simulations is presented in **Table 2**.

**Table 2:** Demographic characteristics of patients enrolled in the AFRINEST and SATT trials (Zaidi et al., 2013b; African Neonatal Sepsis Trial, et al., 2015; Baqui et al., 2015), which were used in the simulation scenarios.

Patient characteristics	Value
Number of patients	10,840
Post-natal age (days), median (range)	16 (1 – 59)
Weight (kg), median (range)	3.3 (1.5 – 8)
Male, %	51
Female, %	49

### Pharmacokinetic Model

As shown in **Figure S2** model parameterization based on first order absorption and two-compartment disposition allowed the characterization of amoxicillin plasma levels in the target paediatric population. One subject was excluded from the analysis due to significantly higher concentrations than the rest of the population. The deviation met the criteria for outlier data handling.

**Figure 3:**
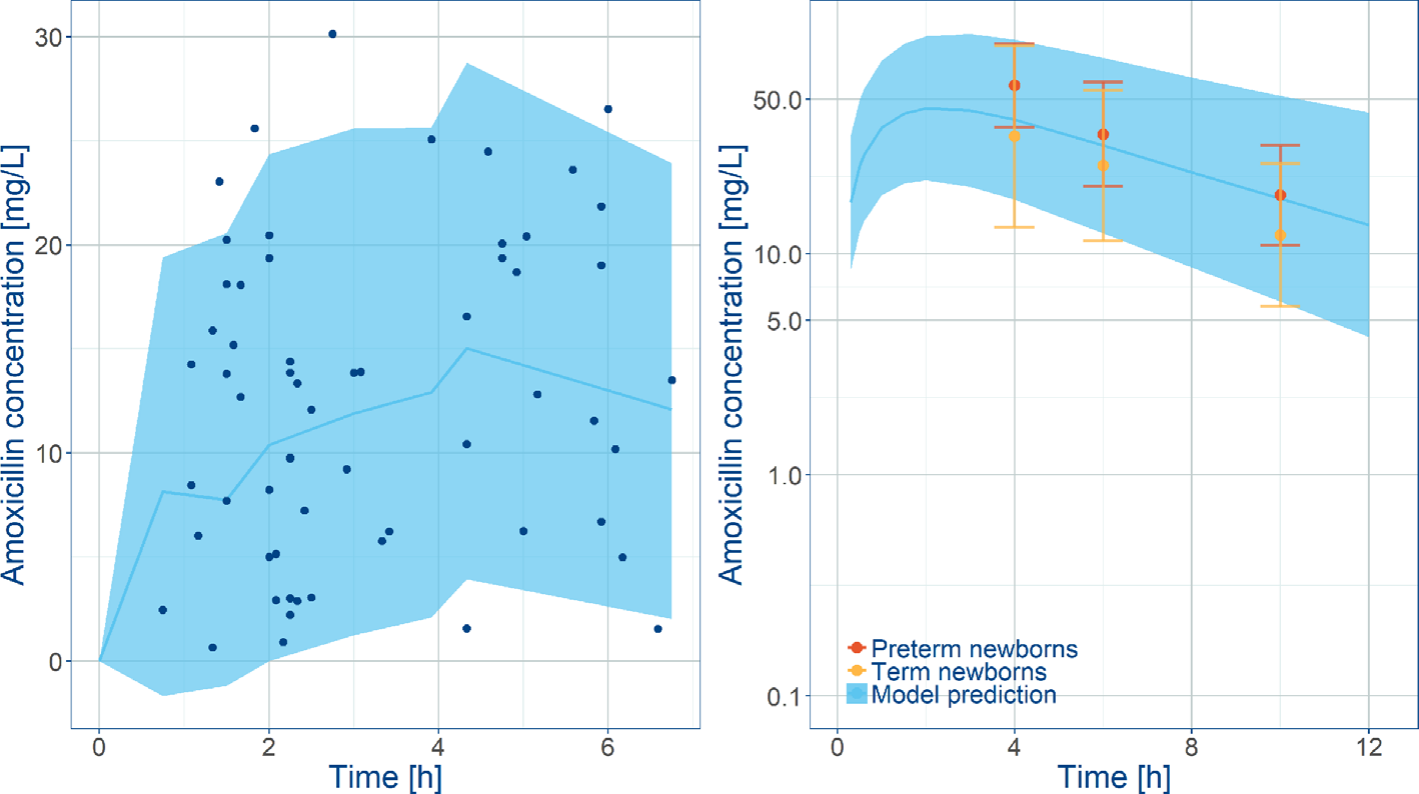
Comparison between model predictions and observed data. Left panel shows VPC along with observed amoxicillin concentrations in the SATT trial (Mir, 2013) after weight‐banded dosing in neonates. Right plot shows model predictions along with observed amoxicillin concentrations in healthy subjects overlaid with observed exposures in pre-term and term newborns diagnosed with other infections. Blue lines represent the predicted median concentrations; shaded areas depict the predicted 5^th^ and 95^th^ percentiles. The error bars indicate the 5^th^ and 95^th^ percentiles the observed data in Weingartner et al., 1977.

Despite the relatively small population, in addition to the effect of covariate factors identified as influential on clearance (i.e. postnatal age, plasma albumin levels, weight at birth) and volume of distribution (i.e. sepsis), it was possible to identify inter-individual variability in clearance and central volume. Model parameters were estimated without significant correlations and with good precision. Goodness-‐of-‐fit plots showed that the model adequately described the data (**Figure S3**), yielding unbiased population and individual predictions. In addition, the visual predictive check reveals that amoxicillin plasma concentrations from neonatal sepsis patients enrolled into the SATT study were approximately 5-fold lower than previously reported with comparable doses in age-matched patients with non-systemic infections (**Figure 3**). An overview of the final model parameters is summarized in **Table 3**. Individual empirical Bayesian *post-hoc* parameter estimates are reported in **Table S1**. It is interesting to highlight that sepsis patients showed a 4-fold increase in the central and peripheral volume of distribution.

**Table 3:** Population pharmacokinetic model parameters and bootstrap estimates used to describe the concentration vs. time profiles of amoxicillin in neonatal sepsis patients (n = 44).

Parameters	Population point estimate	Bootstrap estimate 90%-CI
CL_amik,pop_, amikacin clearance [L/h] Inter-individual variability (CV%)	0.0493 30.0	– 29.8 – 30.2
V_c,pop_, central volume [L] Inter-individual variability (CV%) Inter-occasion variability (CV%)	13.5 38.9 29.1	– 38.2 – 39.2 0.3 – 139
V_p,pop_, peripheral volume [L]	14.1	–
Q_pop_, inter-compartmental clearance [L/h]	3.70	0.36 – 9.02
K_a_, absorption rate constant [h^-1^]	0.244	0.0936 – 0.823
F, bioavailability	0.650	–
Dis_max_, maximum effect of disease on volume of distribution	0.126	0.028 – 0.348
Proportional error (CV%)	22.5	0.225 – 32.5
Additive error [mg/L]	4.94	0.459 – 6.09

Final estimates were obtained by fitting of pharmacokinetic data from patients in the SATT trial (Mir, 2013).

### Predicted Amoxicillin Exposure in the AFRINEST/SATT Studies

**Figure 4:**
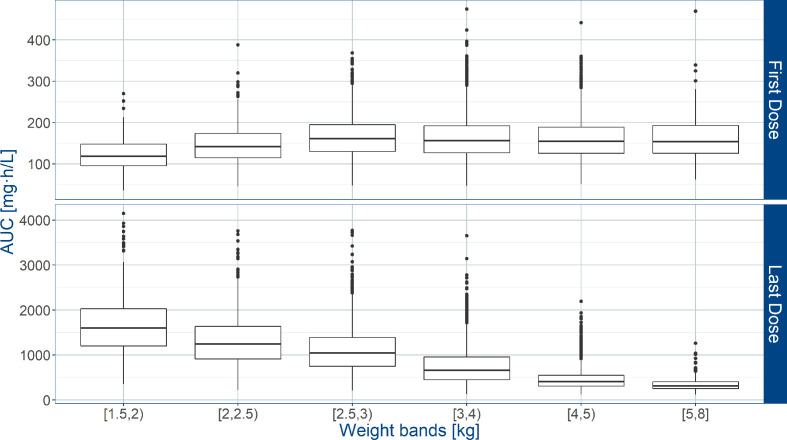
Predicted amoxicillin area under the concentration vs. time curve (AUC) in sepsis patients aged 0 to 59 days. Estimates are summarized according to the weight bands used in the AFRINEST trial protocols. Upper and lower panels show the predictions after the first and last dose, respectively. Hinges represent 25^th^ and 75^th^ percentiles (respectively, Q1 and Q3), whiskers represent Q1 − 1.5IQR and Q3 + 1.5IQR, respectively, where IQR is the interquartile range. All the subjects outside this range are represented by the dots (N= 10840).

Whereas the overall exposure to amoxicillin expressed as AUC and T>MIC, was found to be appropriate across the various weight bands, considerable fluctuation was observed in patients with lower body weight. Significant differences were also predicted between first and last dose (**Figure 4**). Based on the proposed exposure criteria, which aimed at maximizing T>MIC, it can be seen that amoxicillin dosing may be optimized in patients in the lower weight bands, i.e. those with body weight < 4 kg.

**Figure 5:**
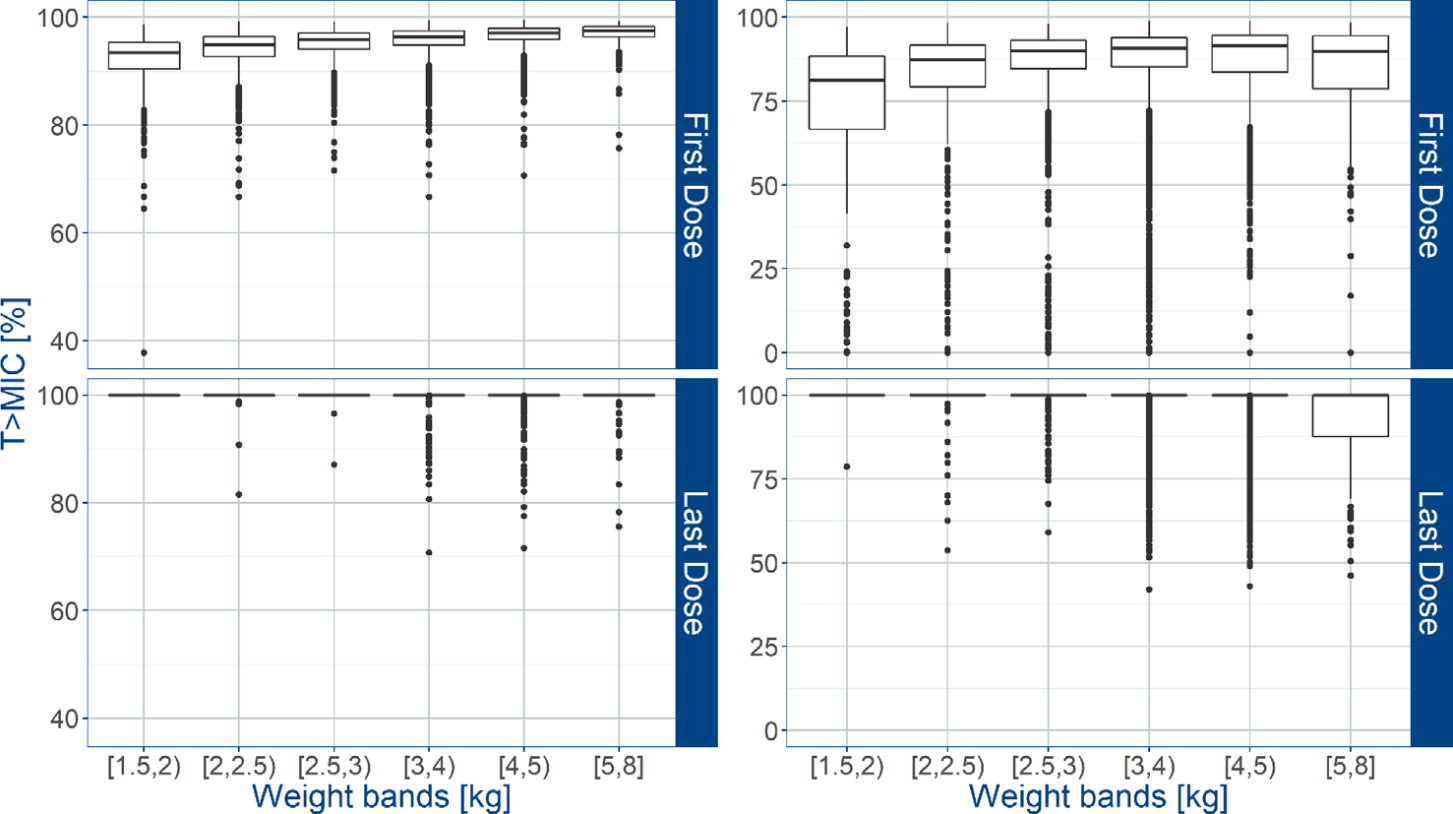
Predicted % of the time above MIC (T>MIC) after administration of amoxicillin in sepsis patients aged 0 to 59 days. Estimates for two different MIC values (4 mg/L on the left; 8 mg/L on the right) are summarized according to the weight bands used in the AFRINEST trial protocols. Upper and lower panels show the predictions after the first and last dose, respectively. Hinges represent 25^th^ and 75^th^ percentiles (respectively, Q1 and Q3), whiskers represent Q1 − 1.5IQR and Q3 + 1.5IQR, respectively, where IQR is the interquartile range. All the subjects outside this range are represented by the dots (N = 10840).

An overview of the variability in the predicted T>MIC observed across the different weight bands, is shown in **Figure 5**, where whisker-box plots depict the distribution of this PKPD index after the first and last dose of amoxicillin, taking into account the treatment cohorts and actual body weight distribution in the study population. As the susceptibility of different strains varies, results are summarized for MIC values of 4 and 8 mg/L. It is clear that when MIC values of 8 mg/L are considered (**Table 4**), T>MIC is lower in a small proportion of patients with low body weight. This is observed despite the use of dosing regimens in mg/kg.

**Table 4:** Predicted percentage (%) of sepsis patients aged 0 – 59 days in the AFRINEST trials with T > MIC (MIC = 8 and 4 mg/L) below the reference threshold level of 50%.

*Trial weight band First dose*	*MIC= 8 mg/L*		*MIC= 4 mg/L*		
	% of patients with T>MIC < 50%	*No. patients/No. patients in weight band*	% of patients with T>MIC < 50%	*No. patients/No. patients in weight band*
*<2 kg*	13.8	51/369	0	0/369
*2–2.5 kg*	4.6	45/977	0	0/977
*2.5–3 kg*	2.5	50/1983	0	0/1983
*3–4 kg*	3.1	152/4926	0	1/4926
*4–5 kg*	2.3	51/2208	0	2/2208
*5–6 kg*	2.1	8/377	0.2	1/377

Data are summarized according to the weight bands used in the trials.

### Comparison Between the Proposed Simplified Regimen and WHO Recommendations

**Figure 6:**
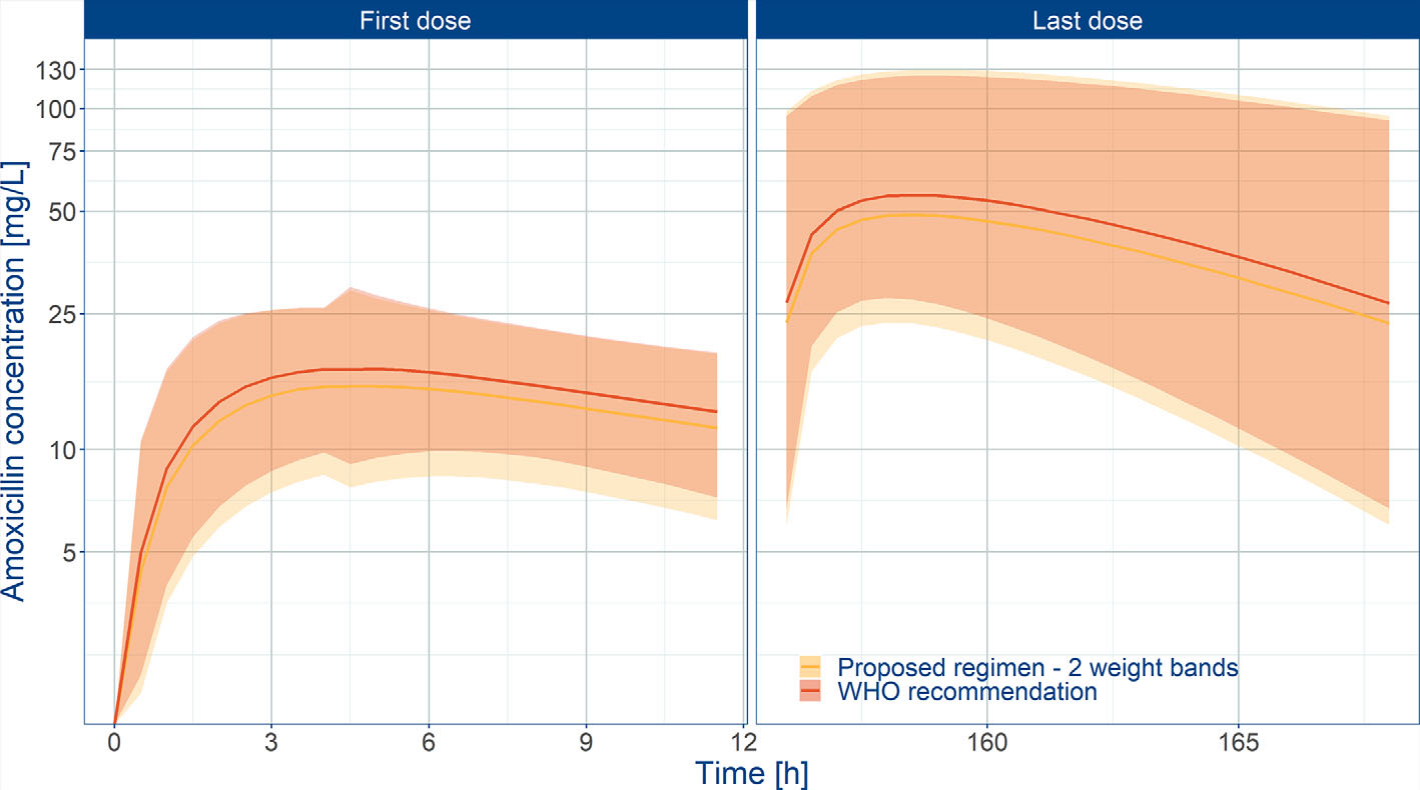
Predicted amoxicillin concentration vs. time profile in sepsis patients aged 0 to 59 days. Panels show how the proposed two-weight banded regimen compares to the 2015 WHO recommended regimen. Solid line depicts the median profile; shaded area represents the 90% prediction intervals. First dose (day 1) and last dose (day 7) are shown to highlight the impact of disease on the pharmacokinetics of amoxicillin. Overall the proposed weight-banded regimen results in similar exposure ranges.

**Figure 7:**
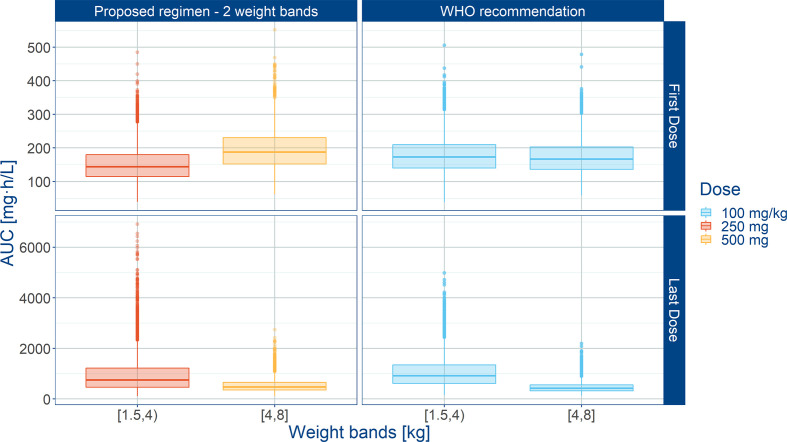
Predicted amoxicillin AUC in sepsis patients aged 0 to 59 days stratified according to a simplified regimen with two weight bands. Panels show how the proposed two-weight banded regimen compares with the 2015 WHO recommended regimen. Hinges represent 25^th^ and 75^th^ percentiles (respectively, Q1 and Q3), whiskers represent Q1 − 1.5IQR and Q3 + 1.5IQR, respectively, where IQR is the inter-quartile range. All the subjects outside this range are represented by the dots. First dose (day 1) and last dose (day 7) are shown to illustrate the effect of disease on the pharmacokinetics of amoxicillin. Legend indicates the total daily dose for a b.i.d. regimen.

Even though the current recommendations by WHO based on a mg/kg dose appear to provide appropriate exposure to amoxicillin in neonates and young infants, our analysis demonstrates the feasibility of implementing a fixed dose regimen in combination with two weight bands, namely: 125 mg b.i.d. for patients with body weight < 4.0 kg and 250 mg b.i.d. for patients with body weight ≥ 4.0 kg. **Figure 6** shows the population predicted plasma concentration vs. time profile of amoxicillin for the proposed simplified regimen along with the 90% confidence intervals, as compared to the WHO recommendations. These profiles correspond to the systemic exposure ranges shown in **Figure 7**. As it can be observed, the two regimens seem to overlap considerably with each other. The main difference between them refers to the lowest weight band (< 4.0 kg), for whom a fixed daily dose of 250 mg is proposed. From a safety perspective the use of a simplified regimen also ensures acceptable peak concentrations, with most patients with amoxicillin levels below 100 mg/L (**Figure S4**).

Another point to consider when comparing treatment scenarios are the differences in drug levels at the end of the dosing interval. Trough values (**Figure S5**) need to warrant antibiotic activity and consequently contribute to the overall T>MIC.

**Figure 8:**
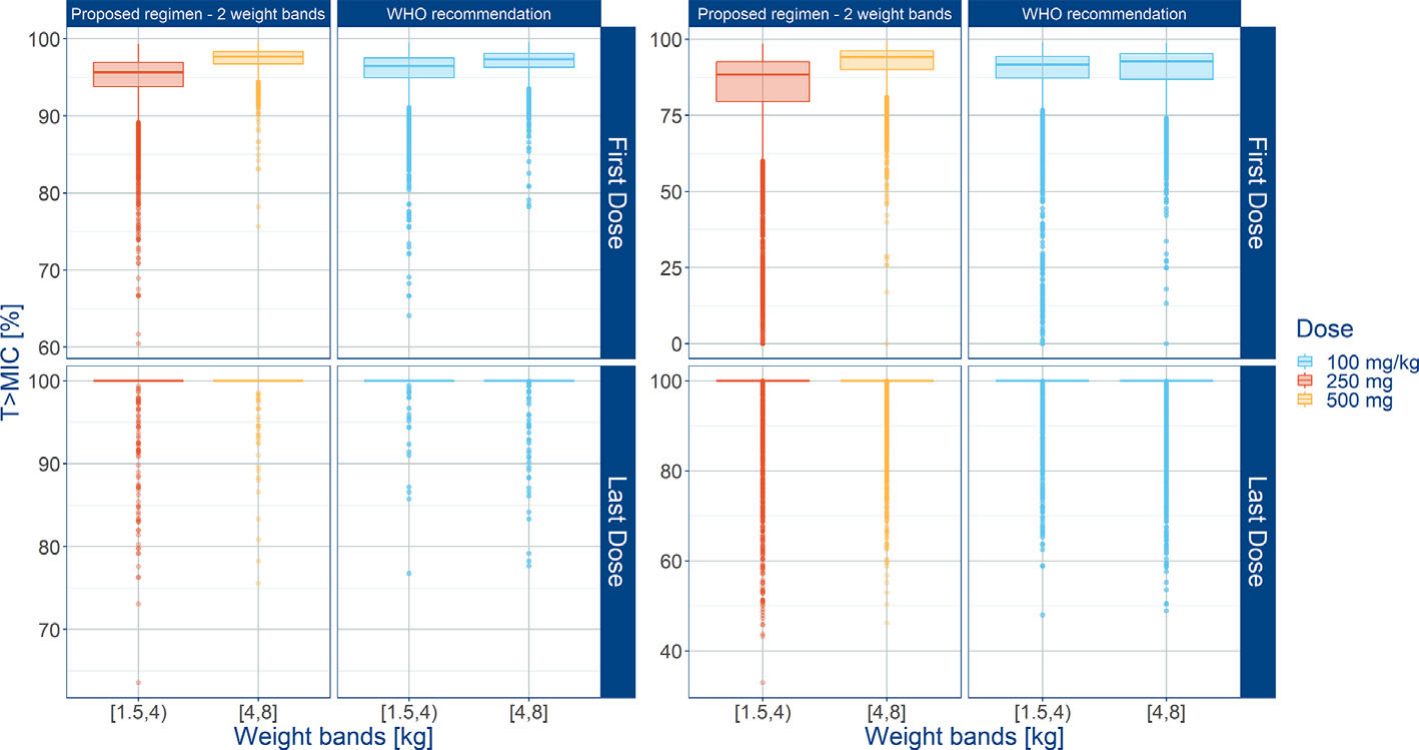
Predicted % of the time above MIC (T>MIC) after administration of amoxicillin to sepsis patients aged 0 to 59 days stratified according to a simplified regimen with two weight bands. Estimates for two different MIC values (4 mg/L on the left; 8 mg/L on the right) are summarized according to the weight bands used in each simulation scenario. Panels show how the proposed two-weight banded regimen compares to the 2015 WHO recommended regimen. Hinges represent 25^th^ and 75^th^ percentiles (respectively, Q1 and Q3), whiskers represent Q1 − 1.5IQR and Q3 + 1.5IQR, respectively, where IQR is the inter-quartile range. All the subjects outside this range are represented by the dots. First dose (day 1) and last dose (day 7) are shown to illustrate the effect of disease on the pharmacokinetics of amoxicillin. Legend indicates the total daily dose for a b.i.d. regimen.

**Figure 9:**
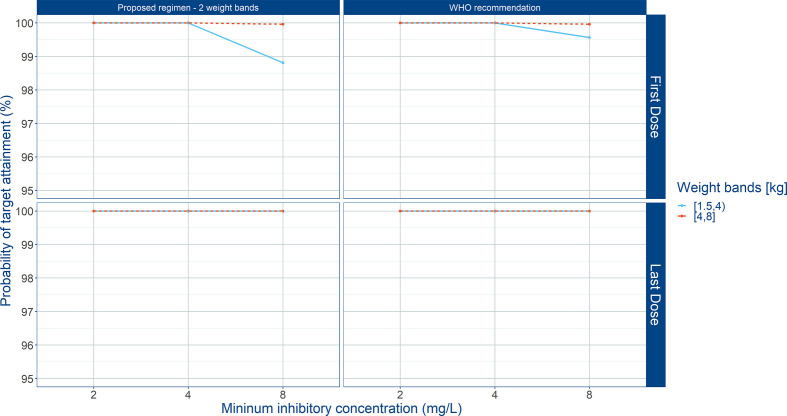
Probability of target attainment (PTA) in sepsis patients aged 0 to 59 days. Panels show how the proposed weight-banded regimen compares to the 2015 WHO recommended dose. Amoxicillin therapeutic target exposure was defined T>MIC ≥ 50%. Three different MIC values (2, 4, and 8 mg/L) were considered. First dose (day 1) and last dose (day 7) are shown to illustrate the effect of disease on the pharmacokinetics of amoxicillin. Legend indicates the total daily dose for a b.i.d. regimen.

Summary statistics of secondary pharmacokinetic parameters, C_max_, C_min_, and AUC are presented along with the 90% confidence intervals in **Table 5**. To ensure accurate assessment of the implications of a simplified regimen relative to the 2015 WHO recommendations, the T>MIC was summarized for different MIC values of 4 and 8 mg/L (**Figure 8**). In addition, the PTA was calculated and is shown in **Figure 9**.

**Table 5:** Predicted exposure to amoxicillin following administration of 250 and 500 mg doses according to a twice daily regimen.

Weight band	C_max_ (mg/L)	C_min_ (mg/L)	AUC (mg·h/L)	T>MIC (h)MIC = 2 mg/L	T>MIC (h)MIC = 4 mg/L	T>MIC (h)MIC = 8 mg/L
< 4 kg	26 (11 – 120)	14 (6 – 80)	254 (93 – 1846)	11.9 (11.5 – 12.0)	11.8 (10.9 – 12.0)	11.4 (7.2 – 12.0)
≥ 4 kg	32 (16 – 75)	12 (6 – 37)	274 (124 – 901)	11.9 (11.7 – 12.0)	11.9 (11.4 – 12.0)	11.6 (9.1 – 12.0)

C_max_, peak concentrations; C_min_, trough concentrations; AUC, area under the concentration vs. time curve; T > MIC, time above the MIC.

Values shown are the mean predictions, 5^th^ and 95^th^ percentiles after the first dose.

## Discussion and Conclusions

While careful use of antibiotics is still needed in all paediatric settings, appropriate drug selection and dose optimization are essential when referral is not feasible. Despite the availability of guidelines for managing possible serious bacterial infection in young infants (Muller-Pebody et al., 2011; World Health Organization, 2015a), limited attention has been given to dose optimization when evidence is generated in support of the benefit-risk profile of a treatment in this vulnerable population, such as AFRINEST and SATT, where empirical regimens have been tested (Zaidi et al., 2013a; Zaidi et al., 2013b; African Neonatal Sepsis Trial et al., 2015; Baqui et al., 2015; Mir et al., 2017).

Recently, Fuchs and colleagues performed a review of the appropriate empirical therapy for treating sepsis in neonates and children (Fuchs et al., 2018). The authors focus on the current WHO guidelines supporting the use of gentamicin and penicillin for hospital-based patients or gentamicin (IM) and amoxicillin (oral) when referral to a hospital is not possible, and suggest that there is no strong evidence to change them. Unfortunately, neither this review nor previous ones have evaluated the feasibility of simplified dosing regimens for amoxicillin in neonatal sepsis patients taking into account PKPD principles. In fact, the most recent WHO guideline (World Health Organization, 2015a) was based on two critical outcomes, namely 1) death within two weeks of starting treatment for PSBI; and 2) treatment failure by day 8 after starting treatment, need for hospitalization/referral, need to change antibiotics, new signs of clinical infection or persistence of signs of illness. Whereas adherence to treatment was also taken into consideration for the selection of the simplest antibiotic regimens that are both safe and effective in children 0–59 days old, it appears that an opportunity has been missed ensure that recommendations are further supported by a scientifically robust dose rationale.

In the current investigation, we have applied quantitative clinical pharmacology methods to identify a simplified regimen for amoxicillin in new-borns and young infants. It has been previously demonstrated that accurate characterization of PKPD relationships allows not only for the selection of the best drug to treat a specific bacterial pathogen, but also enables the optimization of the dosing regimen (Bhavnani et al., 2006; Downes et al., 2014).

### Population Pharmacokinetics and Model Parameteri*z*ation

In spite of the widespread use of amoxicillin in paediatric infections, pharmacokinetic data in neonates with serious bacterial infections are rather sparse (Ginsburg et al., 1979; Rudoy et al., 1979; Huisman-de Boer et al., 1995). In reality, among the recent studies in neonatal sepsis, blood samples for pharmacokinetic analysis have been collected only in the SATT trial (Mir, 2013). This limitation is partly overcome by evolving understanding of paediatric pharmacology, which shows that age (maturation) and weight-related differences in renal function ultimately determine the observed changes in the pharmacokinetics of amoxicillin in neonates. Evidence from other drugs suggests that any attempt to define the dose rationale in this population will also need to consider the contribution of factors such as critical illness, obesity and immune deficiency, which often compound the effect of age and weight (Levison and Levison, 2009; Suzuki et al., 2009; Bellanti and Della Pasqua, 2011).

Surprisingly, the population pharmacokinetics of amoxicillin has never been investigated in neonatal sepsis. As a consequence, we have had to resort to bridging and extrapolation concepts to describe drug exposure in this population. In this context, we have applied an integrated approach to ensure that organ maturation, developmental growth and disease-related factors were considered. While the model developed by Carlier and colleagues (Carlier et al., 2013) allowed us to scale pharmacokinetics taking into account the effect of size on drug disposition, it could not account for the role of maturation or the impact of disease-related changes in vascular permeability and hemodynamics. The use of aminoglycoside clearance as a marker of the renal processes associated with drug elimination represented a practical solution (Zhao et al., 2013). In fact, the work by Zhao et al. reveals the predictive value of amikacin clearance as a marker of renal maturation during the neonatal period. Moreover, the amikacin maturation model enabled reasonable prediction of the clearance of vancomycin, which similarly to amoxicillin, is also excreted by tubular mechanisms (Contrepois et al., 1985; Fanos and Cataldi, 2001). Their results show that prediction bias was not significantly correlated with developmental factors (e.g. age and body weight), indicating that amikacin clearance could be used to predict the dosage regimens of other renally eliminated drugs.

In contrast to most investigations in paediatric clinical pharmacology, where maturation and size are usually explanatory factors for the differences in drug disposition in new-borns and young infants, our analysis also sheds light into the magnitude of the effect of disease-related changes (e.g., electrolyte balance and hemodynamics) on the disposition of amoxicillin. In fact, the impact of sepsis on systemic exposure only became evident after careful evaluation of sparse sampled data from patients in the SATT study. Differently from the findings in critically ill adult patients (Carlier et al., 2013), our results suggest major alteration in the distribution of amoxicillin at the onset of treatment. The observed differences reflect the pathophysiology of serious bacterial infections. Endothelial damage provoked by systemic inflammatory response syndrome (SIRS) may result in an increase in capillary permeability and interstitial edema formation, all of which ultimately affect the volume of distribution (De Paepe et al., 2002; Fuster-Lluch et al., 2008; Vazquez et al., 2008).

### Feasibility of Simplified Dosing Regimens

Based on a statistical model of the demographic characteristics of the target population, in which the correlation between actual body weight, weight at birth and gestational/post-natal age is retained, clinical trial simulations were used to evaluate the impact of different doses and dosing regimens. Using T>MIC as the primary selection criterion along with the corresponding probability of target attainment, our results show that it is possible to treat patients with fixed amoxicillin doses according to two weight bands (< 4.0 and ≥ 4.0 kg), eliminating the complexities of doses in mg/kg. In addition, there is no evidence in the clinical literature to suggest that the proposed dosing regimen would represent an increased risk of renal toxicity (e.g. crystalluria or nephritis) in this population (Moesch et al., 1990; Fogazzi et al., 2003). Hence, the use of weight-banded dosing represents a major opportunity for the treatment of neonatal sepsis in resource-limited settings. It will allow for the use of other dosage forms to be prescribed, including sachets and dispersible paediatric tablets, which may have many potential advantages over traditional suspensions.

We acknowledge the challenges to perform a more comprehensive evaluation of the pharmacokinetics of amoxicillin in community-based settings. Our work illustrates the role of meta-analytical approaches to extrapolate pharmacokinetics across populations with sufficient precision to ensure accurate evaluation of the impact of covariates on drug disposition. We also acknowledge that assumptions had to be made about the role of other intrinsic and extrinsic sources of variability (e.g., compliance, disease severity, age of onset), but these factors should not affect the conclusions drawn from the current analysis.

Undoubtedly, this paper has a few limitations. First, we did not have access to the individual patient isolates to establish whether individual differences in pharmacokinetics could have immediate clinical implications, i.e., how long drug exposure in each patient remained above the MIC and whether patients who showed lowest drug levels correlated with the fraction of patients (4%) who showed positive microbiology results after treatment in these trials. We have relied therefore on established MIC values and inferred pharmacokinetic variability based on observed data and known covariate distributions. From a modeling perspective, the lack of pharmacokinetic data at different days after start of treatment with amoxicillin has forced us to make use of an empirical function to describe the changes in vascular permeability and hemodynamics, which are associated with the onset and progression of SIRS. Given that pharmacokinetic data from neonatal patients with respiratory infection seemed to confirm the predicted parameter distributions in the absence of SIRS (Huisman-de Boer et al., 1995), we have not deemed necessary to perform a formal sensitivity analysis to further assess the effect of fixed parameter estimates on the subsequent simulation scenarios. Lastly, it should be noted that the results reported here were based on total rather than free plasma concentrations. This should not alter the recommendations arising from our analysis, as the mean protein binding for amoxicillin is very low (11.7 + 2.7%) (Pullen et al., 2006). In fact, a preliminary evaluation of the different simulation scenarios using free amoxicillin concentrations showed comparable results for both free and total plasma concentrations.

In summary, a simplified fixed dose regimen based on two weight bands has been identified for amoxicillin, which maximizes the proportion of patients who remain above MIC for most of the dosing interval. This regimen ensures probability of target attainment comparable to levels achieved after a 50 mg/kg dose. Obviously, prospective evaluation of pharmacokinetics in the target population should be considered to confirm model predictions. Such a study could be performed in community-based settings using patient friendly techniques such as dried blood spots.

## Data Availability Statement

The data sets generated for this study are available on request to the corresponding author.

## Ethics Statement

The studies involving human participants were reviewed and approved by the local ethics committees. The ethics committee details and affiliations were previously published by the AFRINEST and SATT study groups. Written informed consent to participate in this study was provided by the participants’ legal guardian/next of kin.

## Author Contributions

SD and FTM contributed to the data analysis, presentation of the results and preparation of the manuscript. ODP contributed to the discussion of the clinical implications and review of the manuscript.

## Funding

This project has received financial support from Save the Children Federation, Inc., Washington, USA.

## Conflict of Interest

The authors declare that the research was conducted in the absence of any commercial or financial relationships that could be construed as a potential conflict of interest.
