# Supplementary material for: Dose Rationale for Amoxicillin in Neonatal Sepsis When Referral Is Not Possible
Source: Front Pharmacol. 2020 Sep 25;11:521933. doi: 10.3389/fphar.2020.521933 (PMC7549385; doi:10.3389/fphar.2020.521933)
Supplement: Supplementary file 1 [file DataSheet_1.pdf]

Table S1. Individual empirical Bayesian *post-hoc* parameter estimates for amoxicillin in neonatal sepsis patients (n=44) stratified by body weight.

**Parameter**	**Median (95% CI)**	
	< 4 kg	≥ 4 kg
$CL_{amox,pop}$ amoxicillin clearance [L/h]	0.138 (0.056 - 0.635)	0.696 (0.31 - 0.972)
$V_{c,pop,h}$ central volume [L] [1]	0.467 (0.273 - 0.838)	0.7 (0.674 - 0.905)
$V_{p,pop,h}$ peripheral volume [L] [1]	0.492 (0.318 - 0.717)	0.799 (0.737 - 0.892)
$V_{c,pop,d}$ central volume [L] [2]	1.978 (1.512 - 3.006)	2.294 (1.958 - 2.917)
$V_{p,pop,d}$ peripheral volume [L] [2]	2.177 (1.995 - 2.41)	2.495 (2.431 - 2.591)
Q_{pop} inter-compartmental clearance [L/h]	0.293 (0.212 - 0.387)	0.419 (0.395 - 0.455)
K_a absorption rate constant [h^{-1}]	0.228	
F bioavailability[3]	0.65	
$E_{d,max}$ effect of disease on volume of distribution	0.126	

[1]Not including the effect of disease, [2]Including the effect of disease

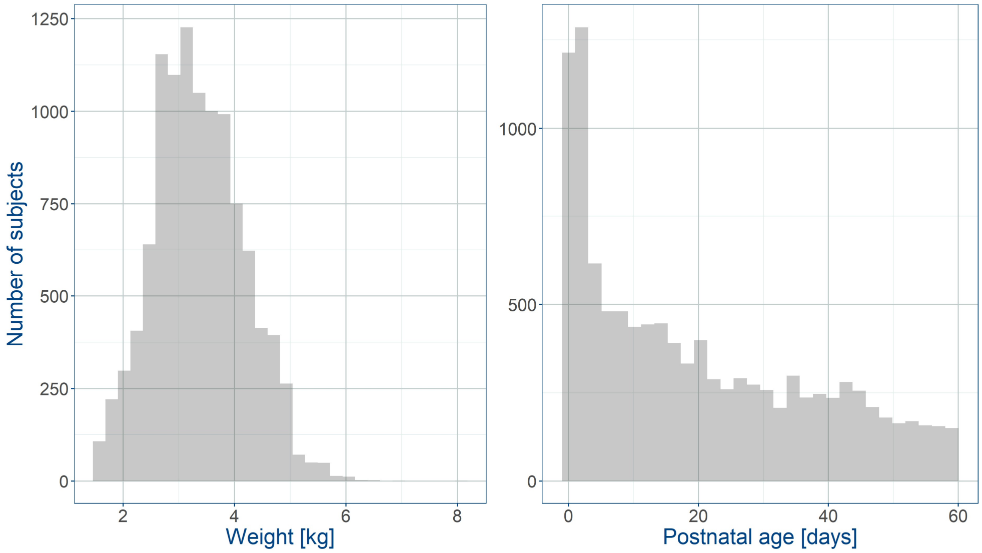

Figure S1. Histograms show weight and postnatal age distributions for the AFRINEST trial population who received amoxicillin for 7 days. Postnatal age and body weight data from the AFRINEST trials were used in conjunction with predicted gestational age to simulate amoxicillin exposure in neonates and young infants with sepsis.

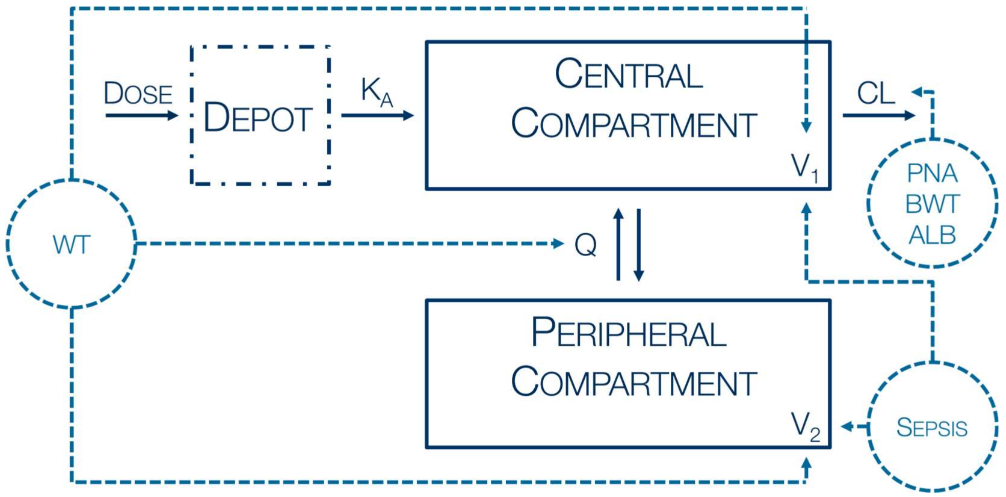

Figure S2. Final population pharmacokinetic model describing the absorption and disposition of amoxicillin in preterm and term neonates. Deterministic parameters included V1 – volume of distribution (central compartment), V2 – volume of distribution (peripheral compartment), Q – inter-compartmental clearance and CL – clearance. Covariate factors identified as influential on clearance were PNA – postnatal age, ALB – plasma albumin levels and BWT – weight at birth. Sepsis was found to affect V1 and V2.

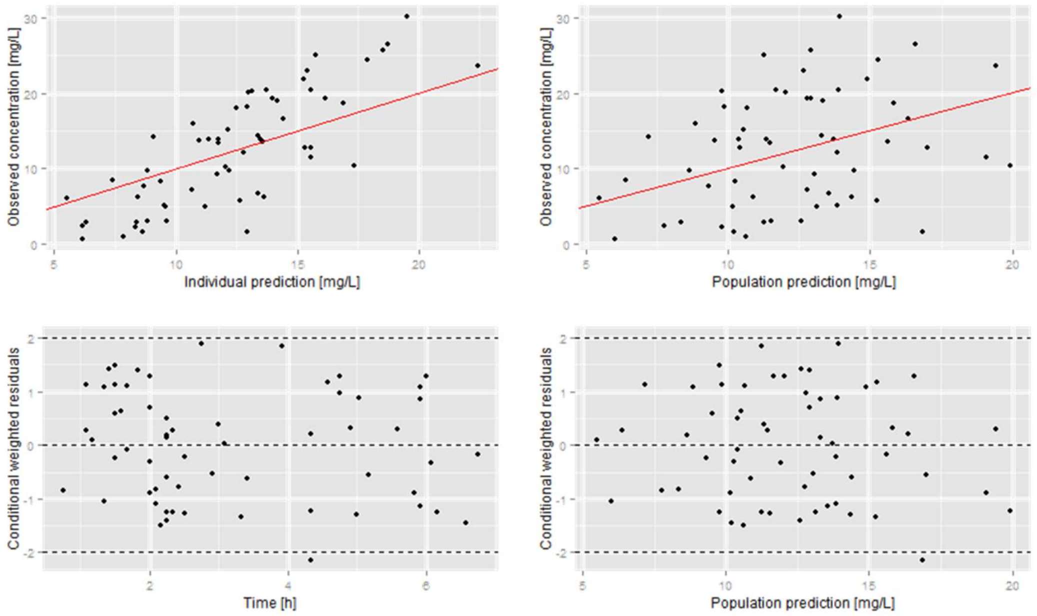

Figure S3. Goodness-of-fit plots for the final pharmacokinetic model. The red line describes the identity line. The dashed lines represent the threshold for the evaluation of conditional weighted residuals.

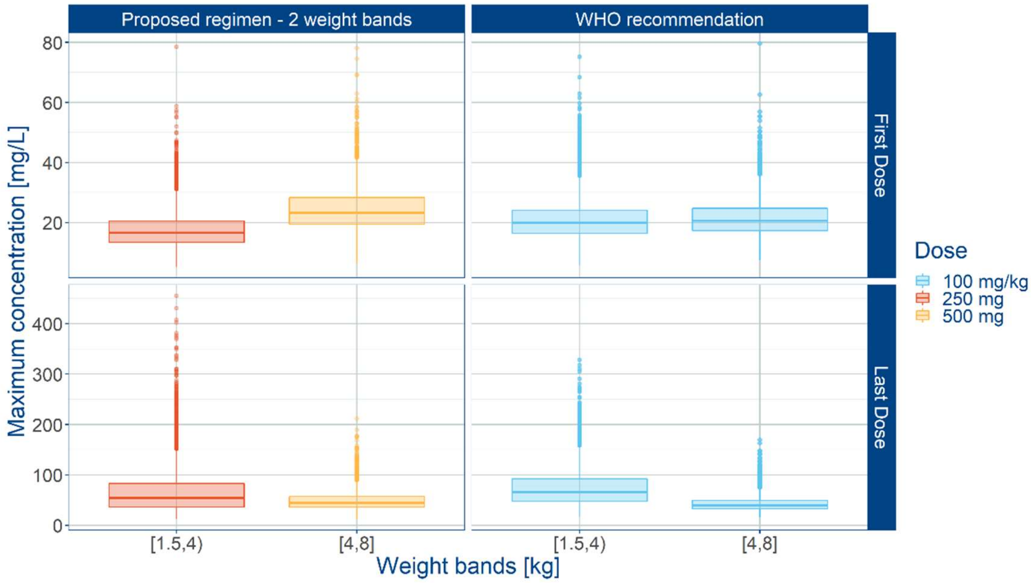

Figure S4. Predicted amoxicillin peak concentrations in sepsis patients aged 0 – 59 days stratified according to a simplified regimen with two weight bands. Panels show how the proposed regimen compares to the 2015 WHO recommended dose (i.e., 50 mg/kg). Nephrotoxicity and crystalluria have been associated with amoxicillin levels higher than 100 mg/L. Hinges represent 25thand 75th percentiles (respectively, Q1 and Q3), whiskers represent Q1 – 1.5IQR and Q3 + 1.5IQR, respectively, where IQR is the interquartile range. All the subjects outside this range are represented by the dots (N= 10840). Legend indicates the total daily dose for a b.i.d. regimen.

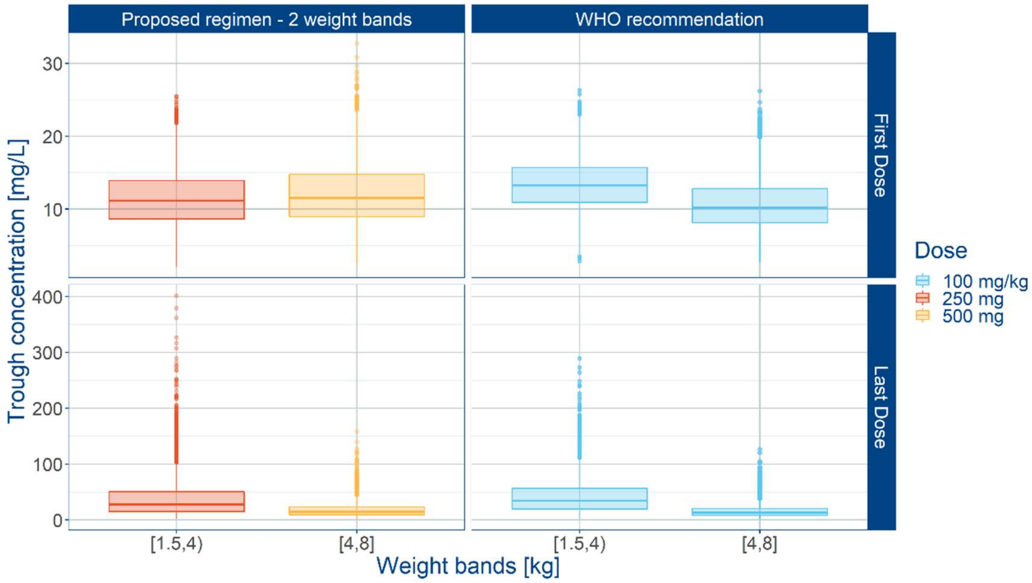

Figure S5. Predicted amoxicillin trough concentrations in sepsis patients aged 0 – 59 days stratified according to a simplified regimen with two weight bands. Panels show how the proposed two-weight banded regimen compares to the 2015 WHO recommended dose (i.e., 50 mg/kg). Hinges represent 25th and 75th percentiles (respectively, Q1 and Q3), whiskers represent Q1 – 1.5IQR and Q3 + 1.5IQR, respectively, where IQR is the inter-quartile range. All the subjects outside this range are represented by the dots (N=10840). First dose (day 1) and last dose (day 7) are shown to illustrate the effect of disease on the pharmacokinetics of amoxicillin. Legend indicates the total daily dose for a b.i.d. regimen.
